# New York County

**Published:** 1899-04

**Authors:** Robert W. Ellis

**Affiliations:** Secretary


Secretary.

### VETERINARY MEDICAL ASSOCIATION OF NEW YORK COUNTY.

THE regular monthly meeting of the Association was called to order at 8.30 P.M., March 1, 1899, President Robertson in the chair. The following members were present at roll-call: Drs. Amling, Bretherton, Bell, J. S. Cattanach, Jr., Clayton, Delaney, Ellis, Gill, Grenside, Goubeaud, Hanson, Keller, O'Shea, and Robertson. Among the visitors present were Prof. W. L. Williams, New York State College; Dr. Howe, of Ohio; and Dr. DuBois of New York City. The minutes of the previous meeting were read and approved.

Dr. C. E. Clayton, Chairman of the Board of Censors, reported that the board held an application for membership from Cyrus H. DuBois, D.V.S., graduate of American Veterinary College, class of 1896; but as a quorum was not present action upon it would have to be deferred until the next meeting. Report was accepted.

Dr. Clayton then read a paper entitled "Median Neurectomy." After listening to Dr. Clayton's paper it was regularly moved and seconded that the By-Laws be suspended, and the visitors have the privilege of the floor in the discussion; carried. The discussion was opened by Dr. Bell, who introduced Prof. Williams, of Ithaca, who indulged in a lengthy discussion of the operation described in Dr. Clayton's paper.

Dr. Howe, of Ohio, was requested by the chair to engage in the discussion, but stated that he had never performed the operation or seen it performed, but gave his results from low plantar neurotomy, which he stated were most satisfactory, and that he had performed it on a great number of horses, giving it precedence over high plantar. While the discussion by the members was animated and interesting, it could not become very general, as but few present had performed the operation. Moved and seconded, that a vote of thanks be extended to Dr. Clayton for his most excellent essay; carried.

Dr. O'Shea, chairman of the Committee on Legislation, reported that two bills to amend the present State law and reopen the registration-books to quacks had already been passed in the Assembly and gone to the Senate, and a third was still in the Assembly. He also called the attention of the Association to bill No. 650, entitled "An act in relation to the establishment of a State livestock sanitary commission, and to provide for the control and suppression of tuberculosis and other dangerous diseases of domestic animals." He stated that the committee desired to get the sense of the meeting as to whether it approved or disapproved of the bill as a whole or any feature in the bill. The discussion which followed brought out the fact that the appointments on the commission, which included two veterinarians, were to be made by the Governor.

After free discussion of the various features of the bill it was moved by Dr. Clayton that the Association approve the bill after amending it so as to make all appointments therein subject to civil-service examination. Seconded and carried.

Moved and seconded, that the meeting adjourn; carried.

Robert W. Ellis, D.V.S.,
Secretary.

